# Impact of ivabradine in decompensated heart failure due to cancer therapy‐related cardiac dysfunction

**DOI:** 10.1002/ccr3.4133

**Published:** 2021-05-05

**Authors:** Yusuke Nakano, Hirohiko Ando, Wataru Suzuki, Hirofumi Ohashi, Yasushi Suzuki, Hiroaki Takashima, Tetsuya Amano

**Affiliations:** ^1^ Department of Cardiology Aichi Medical University Nagakute Japan

**Keywords:** A wave, anthracycline, cancer, CTRCD, decompensated heart failure, E wave, echocardiography, ivabradine

## Abstract

In cases of decompensated heart failure related to cancer therapy‐related cardiac dysfunction, ivabradine administration could lead to an increased stroke volume by reducing the sinus heart rate, resulting in favorable hemodynamics. Assessment of the overlap between the E‐ and A‐waves facilitates understanding the effects of ivabradine in such cases.

## Graphical abstract

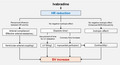

## 1 INTRODUCTION

The incidence of heart failure (HF) due to cancer therapy‐related cardiac dysfunction (CTRCD) is increasing and is associated with poor clinical outcomes.^1^ CTRCD, defined based on reduction in the left ventricular ejection fraction (LVEF), is a well‐documented side effect of cancer therapeutic agents, especially anthracyclines.^2^ Conservative treatment with medications is often difficult, especially in advanced HF.^3^, ^4^ Ivabradine, a specific I_f_ channel inhibitor, lowers the sinus heart rate (HR) without affecting myocardial contractility. It is indicated for controlling HR as a second‐line medication after the optimal therapy with beta‐blocker, angiotensin‐converting enzyme inhibitor (or angiotensin receptor blocker), and mineralocorticoid receptor antagonist in patients with chronic HF and reduced ejection fraction.^5^, ^6^ However, some studies report that ivabradine has a positive effect on the hemodynamics of decompensated HF.^7^, ^8^, ^9^, ^10^ Here, we report a case of advanced HF due to CTRCD refractory to conventional HF therapy that was successfully treated with ivabradine.

## 2 CASE HISTORY / EXAMINATION

A 61‐year‐old man with a history of coronary intervention for angina pectoris, complaining of gradual onset of shortness of breath for 5 days, was admitted to our university hospital for decompensated HF. He reported no cardiovascular risk factors, including hypertension, dyslipidemia, diabetes, obesity, and chronic kidney disease, except for a past habit of smoking. However, he had been diagnosed with stage II diffuse large B‐cell lymphoma with performance status 2 and had been receiving chemotherapies including anthracycline since 2 years. The treatment comprised combination chemotherapy with R‐THP‐COP (rituximab plus tetrahydropyranyladriamycin (pirarubicin, THP: total 400 mg/m^2^), cyclophosphamide (total 6000 mg/m^2^), vincristine, and prednisolone) had been administered every 3 weeks for eight cycles. Computed tomography and positron‐emission tomography had confirmed complete remission. However, because the lymphoma had recurred 10 months later, R‐DeVIC therapy (rituximab combined with etoposide, dexamethasone, ifosfamide (total 3.4 g/m^2^), and carboplatin) had been added every 4 weeks for three cycles. Subsequently, computed tomography had confirmed remission. His cardiac function had gradually declined from the normal limit of left ventricular ejection fraction (LVEF) of 60.0% at baseline to minimum 15.4% on eighteen months after the first initiation of chemotherapy. He had no coronary stenosis on coronary angiography; during his previous hospital admission, and he had been diagnosed with CTRCD according to the diagnostic criteria based on the expert consensus from the American Society of Echocardiography and the European Association of Cardiovascular Imaging.^2^ This hospitalization for HF was the third in the previous 10 months.

On admission, his blood pressure was 96/64 mm Hg, pulse rate 118 /min, body temperature 36.0°C, and oxygen saturation 92% on room air, which increased to 98% with 4 L/min cannula oxygen supply. Physical examination revealed an engorged jugular vein, tachycardia with a grade II systolic murmur over the apex, rales in both lower lung fields, and bilateral edema in the lower extremities.

## 3 INVESTIGATIONS AND TREATMENT

**FIGURE 1:**
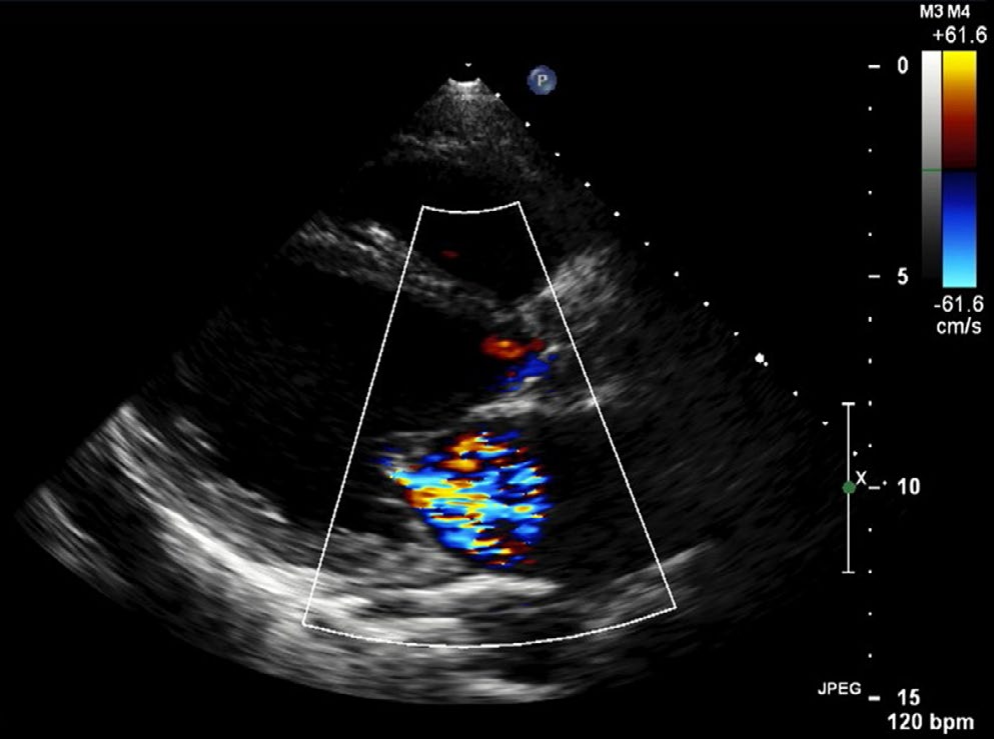
Long‐axis view on echocardiography on admission. Echocardiography showed diffuse severe hypokinesis, increased left ventricular end‐diastolic diameter, and moderate mitral regurgitation

Routine laboratory tests showed an increase in B‐type natriuretic peptide (BNP: 2208 pg/mL), total bilirubin (1.98 mg/dL), aspartate aminotransferase (AST: 81 U/L), alanine aminotransferase (ALT: 75 U/L), blood urea nitrogen (33.1 mg/dL), and creatinine (1.46 mg/dL). Chest radiography revealed cardiomegaly with pulmonary congestion. Electrocardiography demonstrated sinus tachycardia (HR: 104 /min). Transthoracic echocardiography showed diffuse severe hypokinesis of LVEF 30.8%, an increased left ventricular end‐diastolic diameter of 64.4 mm, and moderate mitral regurgitation (Figure 1).

## 4 OUTCOME AND FOLLOW‐UP

**FIGURE 2:**
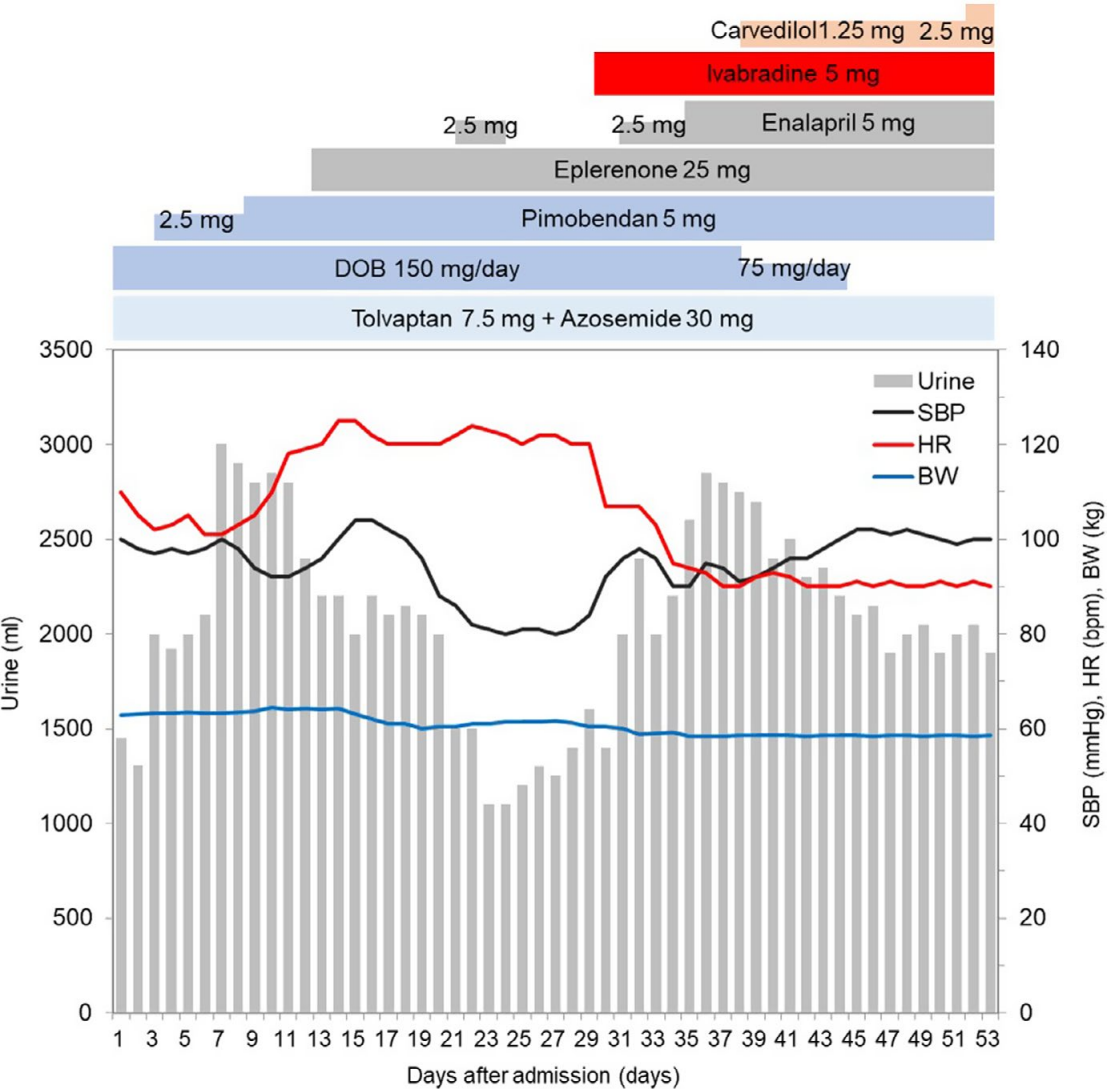
Clinical course. Dobutamine and pimobendane promoted adequate diuresis at the beginning, but gradually result in poor diuresis with HR elevation (120 bpm). After oral ivabradine, the HR decreased and blood pressure increased. SBP, systolic blood pressure; HR, heart rate; BW, body weight

**FIGURE 3:**
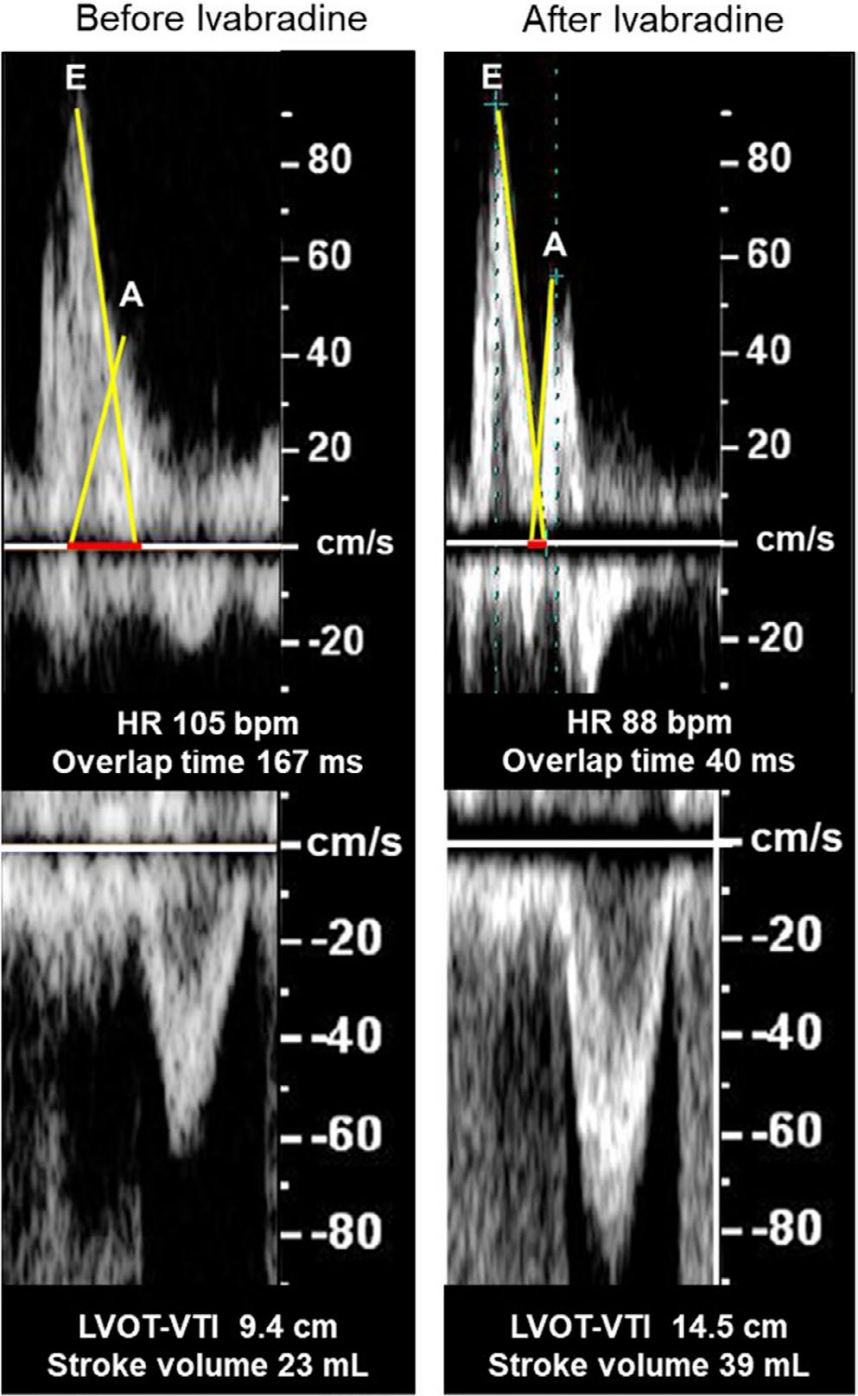
Echocardiographic changes before (left) and after (right) ivabradine administration. Before ivabradine, echocardiography shows an extreme overlap between the E‐ and A‐waves, low LVOT‐VTI, and low SV. After ivabradine, the overlap decreased and LVOT‐VTI and SV increased. HR; heart rate, LVOT‐VTI; velocity‐time integral at the left ventricular outflow tract, SV, stroke volume

**FIGURE 4:**
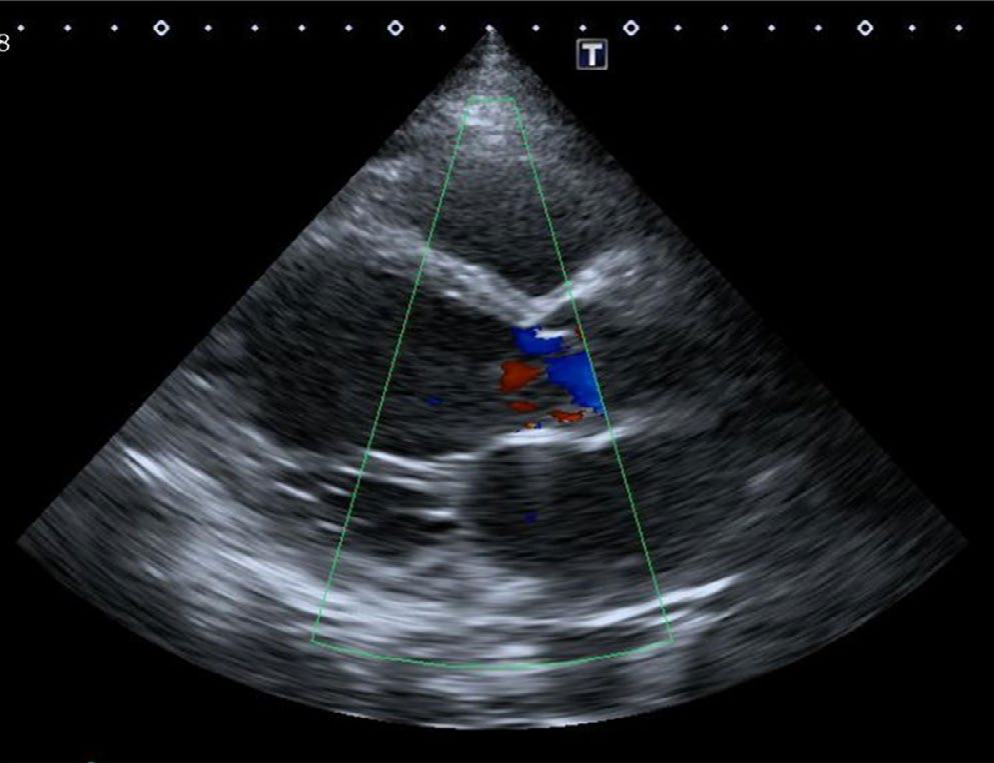
Long‐axis view on echocardiography at the time of discharge. Echocardiography showed diffuse severe hypokinesis, but LVEF and mitral regurgitation had improved compared to the time of admission

As shown in Figure 2, administration of dobutamine and pimobendane in addition to azosemide and tolvaptan resulted in good diuresis initially, which gradually deteriorated and the patient's HR showed an elevation to 120 /min. The subsequent administration of eplerenone and enalapril to manage residual pleural effusion resulted in hemodynamic deterioration with low systolic pressure (approximately 80 mm Hg). Transthoracic echocardiographic examination revealed low stroke volume (SV) and an extreme overlap between the E‐ and A‐waves (Figure 3‐left). Administration of oral ivabradine (5 mg twice daily) improved tachycardia and decreased the overlap; the velocity‐time integral at the left ventricular outflow tract (LVOT‐VTI), SV, and blood pressure increased (Figure 2, and Figure 3‐right). His hemodynamics stabilized subsequently, and the pulmonary congestion disappeared, allowing the patient to be weaned from catecholamines. Eventually, the LVEF increased to 32.0% (Figure 4). His BNP, total bilirubin, AST, ALT, and creatinine levels decreased to 43 pg/mL, 0.54 mg/dL, 37 U/L, 49 U/L, and 0.98 mg/dL, respectively. HF improved, and he was discharged on the 60th day. There was no further worsening of the HF after discharge, but the patient died a few months later because of an intracranial recurrence of lymphoma.

This case report has been published with the prior consent of the patient.

## 5 DISCUSSION

The present case shows the favorable hemodynamic effect of ivabradine in a patient with decompensated HF due to CTRCD, refractory to conventional medical therapy.

It is reported that the incidence of CTRCD due to anthracycline is 9%, with 98% of the cases developing within 1 year. Anthracycline‐induced myocardial damage is irreversible,^11^ and the 2‐year survival rate has been reported to be 50% or less when anthracyclines are the cause of HF. The treatment is often difficult in such cases, where HF is advanced and refractory to inotropes.^3^ The use of ivabradine in decompensated HF is off‐label. However, due to its unique mechanism of reduction of HR without influencing myocardial contraction, ivabradine does not cause the undesired effect of hypotension, which is a major problem on up‐titration of beta‐blockers, and might have favorable effects on hemodynamics even in decompensated HF due to CTRCD. In some patients in advanced HF or cardiogenic shock, ivabradine was safely used to reduce HR and increase SV without impairing their hemodynamic status.^7^, ^9^

**FIGURE 5:**
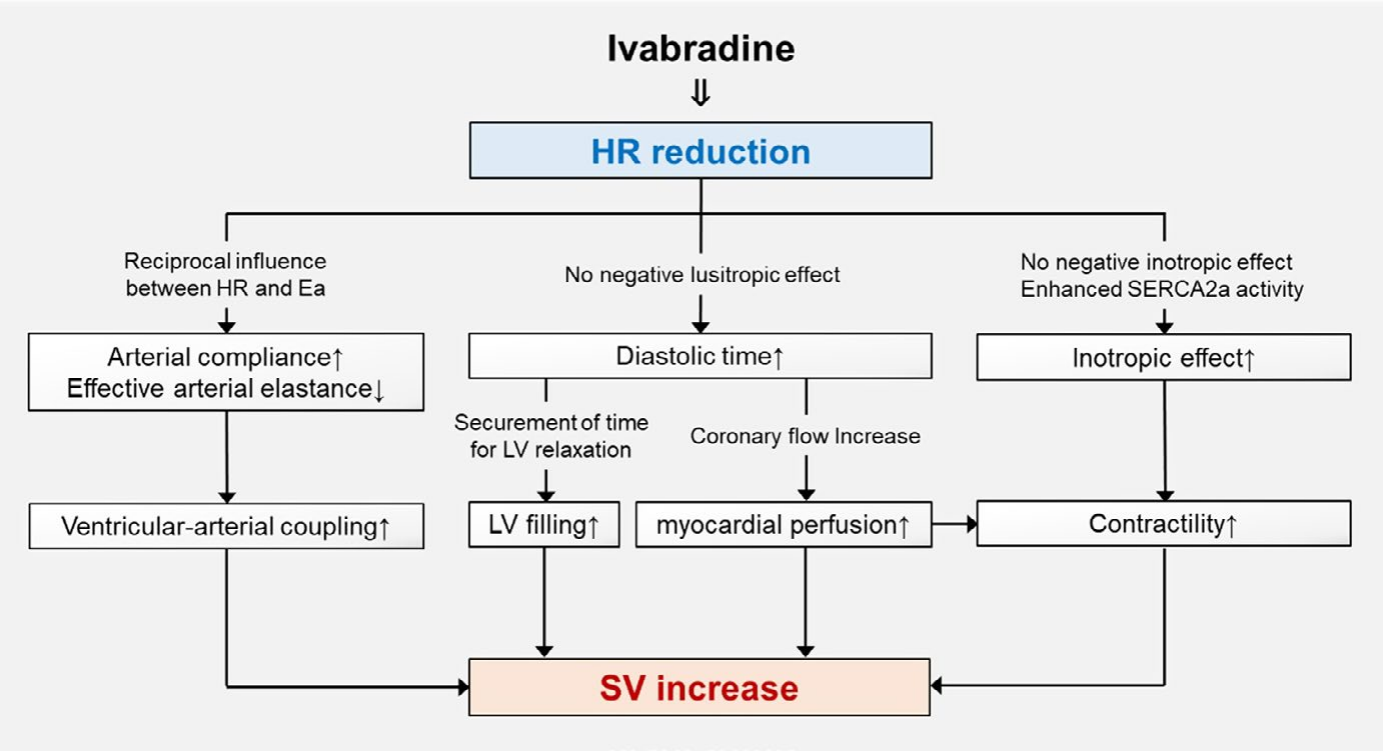
The plausible mechanism of an increase of SV by ivabradine. The increased SV by ivabradine is derived from alterations in hemodynamics such as pre‐ and afterload and myocardial contractility. HR, heart rate; SV, stroke volume; SERCA2a, sarcoplasmic/endoplasmic reticulum calcium ATPase 2a

Although the reason ivabradine increases SV in decompensated HF is not fully understood, the mechanisms are suggested to be as follows (Figure 5). Ivabradine causes prolongation of diastolic time, which leads to an increase of LV diastolic filling along with an increase of coronary perfusion, increasing SV via the Franck‐Starling mechanism.^7^, ^12^, ^13^, ^14^ Moreover, ivabradine has a positive inotropic effect primarily due to increased sarcoplasmic/endoplasmic reticulum calcium ATPase 2a activity and does not have a negative inotropic effect, unlike β‐blockers.^15^ Afterload reduction based on the reciprocal interaction between HR and effective arterial elastance (Ea) has also been reported.^7^, ^12^, ^14^ Ventricular arterial coupling was improved because of the decrease in Ea, resulting in higher SV in patients treated with ivabradine.^14^

**FIGURE 6:**
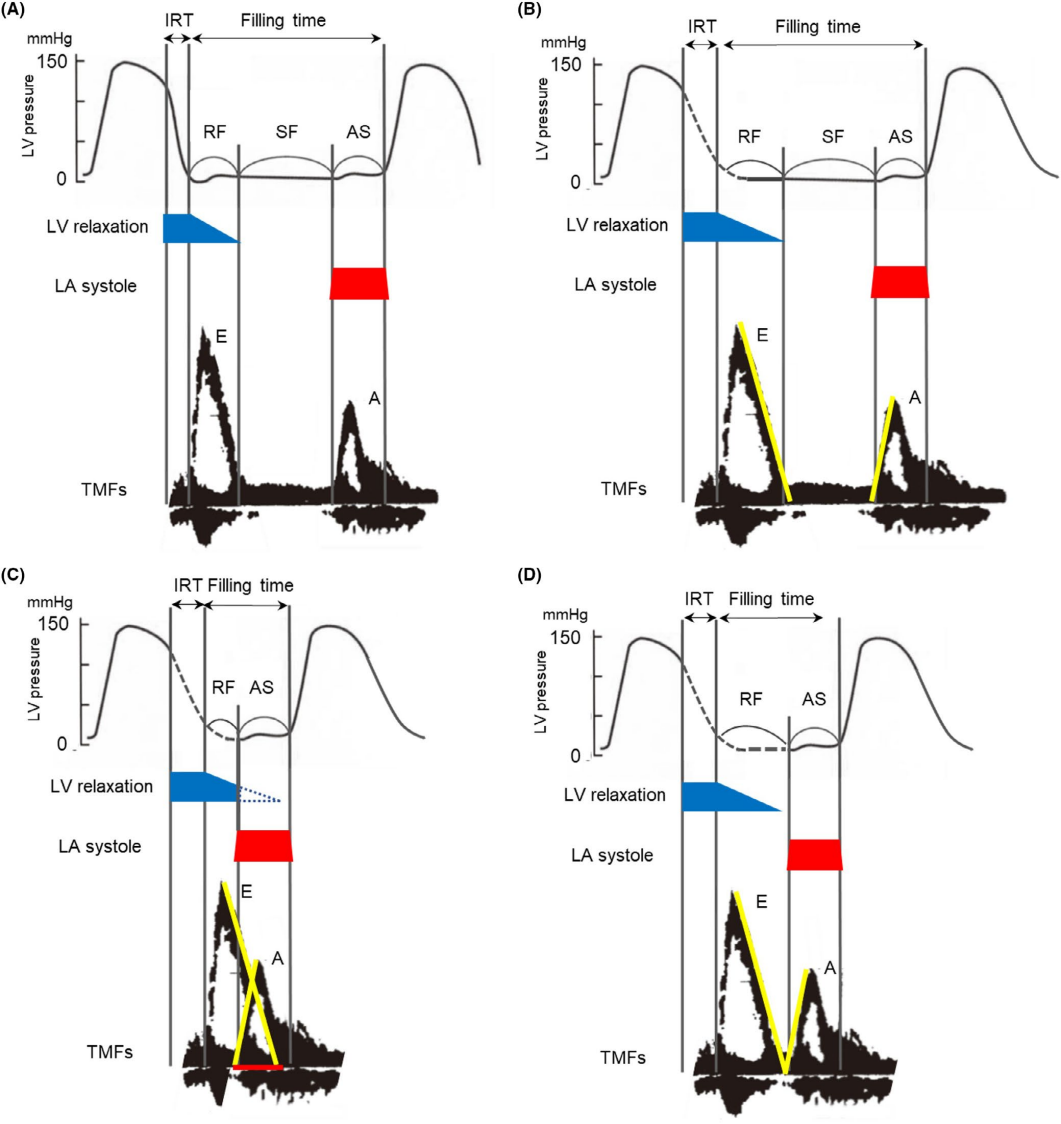
HR optimization from the viewpoint of echocardiography. A, Normal. B, LV diastolic dysfunction prolongs LV relaxation time. C, In higher heart rate, LA systole could interfere with LV complete relaxation. D, Prevention of the E‐ and A‐waves from overlapping using echocardiographic monitoring might help LV complete relaxation. LV, left ventricle; LA, left atrial; TMF, transmitral flow; IRT, isovolumic relaxation time; RF, rapid filling; SF, slow filling; AS, atrial systole; the red line indicates the overlap between the E‐ and A‐waves

How much should the heart rate be reduced by ivabradine? From the viewpoint of echocardiography, monitoring the degree of overlap between the E‐ and A‐waves might be useful as a way to optimize HR (Figure 6). The overlap between E‐ and A‐waves observed at higher sinus HR suggests that LV relaxation is interfered with by atrial systole. This could result in reduced LV filling volume. Izumida et al propose a novel formula to estimate ideal HR in patients with HF using echocardiographic parameters, the overlap between the E‐ and A‐waves, and deceleration time.^16^

In the present case, the HR lowering effect of ivabradine could increase SV, with its attendant favorable hemodynamic consequences. Though medical therapy is often difficult in advanced HF related to CTRCD, ivabradine might be effective as a complementary medical option in these cases. Moreover, assessing the degree of overlap between the E‐ and A‐waves facilitates estimation of the effects of ivabradine.

In conclusion, the administration of ivabradine could lead to improvement of hemodynamic status in decompensated HF related to CTRCD via the increase of SV. Assessment of the overlap between the E‐ and A‐waves facilitates receiving the benefits of ivabradine in such cases. Further large‐sample verification will be needed to confirm these findings.

## CONFLICT OF INTEREST

YN receives lecture fees from Ono Yakuhin. The remaining authors have no disclosures to report.

## AUTHOR CONTRIBUTION

YN: involved in analysis and interpretation of patient's data, conception and design, drafting of the manuscript. HA: involved in conception and design, critical feedback, and revision of the manuscript. WS: involved in the revision of the manuscript with a focus on the echocardiographic aspects. HO, YS, and HT: involved in critical feedback and revision of the manuscript. TA: involved in the revision and final approval of the manuscript. All authors discussed the case and commented on the manuscript at all stages.

## ETHICAL APPROVAL

The Ethics Committee of Aichi Medical University granted an exemption from ethics approval.

## Data Availability

All data generated or analyzed during this study are available as part of the article, and no additional source data are required.

## References

[1] Armenian SH , Lacchetti C , Barac A , et al. Prevention and monitoring of cardiac dysfunction in survivors of adult cancers: american society of clinical oncology clinical practice guideline. J Clin Oncol. 2017;35(8):893‐911. 27918725 10.1200/JCO.2016.70.5400

[2] Plana JC , Galderisi M , Barac A , et al. Expert consensus for multimodality imaging evaluation of adult patients during and after cancer therapy: a report from the American Society of Echocardiography and the European Association of Cardiovascular Imaging. J Am Soc Echocardiogr. 2014;27(9):911‐939. 25172399 10.1016/j.echo.2014.07.012

[3] Felker GM , Thompson RE , Hare JM , et al. Underlying causes and long‐term survival in patients with initially unexplained cardiomyopathy. N Engl J Med. 2000;342(15):1077‐1084. 10760308 10.1056/NEJM200004133421502

[4] Avila MS , Siqueira SRR , Ferreira SMA , Bocchi EA . Prevention and treatment of chemotherapy‐induced cardiotoxicity. Methodist Debakey Cardiovasc J. 2019;15(4):267‐273. 31988687 10.14797/mdcj-15-4-267 PMC6977564

[5] Cowie MR , Anker SD , Cleland JGF , et al. Improving care for patients with acute heart failure: before, during and after hospitalization. ESC heart failure. 2014;1(2):110‐145. 28834628 10.1002/ehf2.12021

[6] Yancy CW , Jessup M , Bozkurt B , et al. 2017 ACC/AHA/HFSA Focused Update of the 2013 ACCF/AHA guideline for the management of heart failure: a report of the American College of Cardiology/American Heart Association task force on clinical practice guidelines and the heart failure society of America. J Card Fail. 2017;23(8):628‐651. 28461259 10.1016/j.cardfail.2017.04.014

[7] De Ferrari GM , Mazzuero A , Agnesina L , et al. Favourable effects of heart rate reduction with intravenous administration of ivabradine in patients with advanced heart failure. Eur J Heart Fail. 2008;10(6):550‐555. 18486549 10.1016/j.ejheart.2008.04.005

[8] Franke J , Schmahl D , Lehrke S , et al. Adjuvant use of ivabradine in acute heart failure due to myocarditis. Case Rep Med. 2011;2011:203690. 21966292 10.1155/2011/203690 PMC3180827

[9] Chiu MH , Howlett JG , Sharma NC . Initiation of ivabradine in cardiogenic shock. ESC heart failure. 2019;6(5):1088‐1091. 31332966 10.1002/ehf2.12499 PMC6816060

[10] Izco MP , Ramirez‐Carracedo R , Navarro IH , et al. Ivabradine in acute heart failure: Effects on heart rate and hemodynamic parameters in a randomized and controlled swine trial. Cardiol J. 2020;27(1):62‐71. 30155868 10.5603/CJ.a2018.0078 PMC8086495

[11] Cardinale D , Colombo A , Bacchiani G , et al. Early detection of anthracycline cardiotoxicity and improvement with heart failure therapy. Circulation. 2015;131(22):1981‐1988. 25948538 10.1161/CIRCULATIONAHA.114.013777

[12] Mulder P , Barbier S , Chagraoui A , et al. Long‐term heart rate reduction induced by the selective I(f) current inhibitor ivabradine improves left ventricular function and intrinsic myocardial structure in congestive heart failure. Circulation. 2004;109(13):1674‐1679. 14981003 10.1161/01.CIR.0000118464.48959.1C

[13] Custodis F , Schirmer SH , Baumhakel M , Heusch G , Bohm M , Laufs U . Vascular pathophysiology in response to increased heart rate. J Am Coll Cardiol. 2010;56(24):1973‐1983. 21126638 10.1016/j.jacc.2010.09.014

[14] Reil JC , Tardif JC , Ford I , et al. Selective heart rate reduction with ivabradine unloads the left ventricle in heart failure patients. J Am Coll Cardiol. 2013;62(21):1977‐1985. 23933545 10.1016/j.jacc.2013.07.027

[15] Xie M , Huang HL , Zhang WH , et al. Increased sarcoplasmic/endoplasmic reticulum calcium ATPase 2a activity underlies the mechanism of the positive inotropic effect of ivabradine. Exp Physiol. 2020;105(3):477‐488. 31912915 10.1113/EP087964

[16] Izumida T , Imamura T , Nakamura M , Fukuda N , Kinugawa K . How to consider target heart rate in patients with systolic heart failure. ESC heart failure. 2020;7(5):3231‐3234. 32592292 10.1002/ehf2.12814 PMC7524252

